# Knowledge, Attitudes, and Practices Towards Dehydration Among Adults in the United Arab Emirates: A Cross-Sectional Study

**DOI:** 10.7759/cureus.79240

**Published:** 2025-02-18

**Authors:** Ahmad Altelly, Amna Lootah, Batoul Daher, Ghaith Alsabbagh, Ranim Alsabbagh, Mohammad Zaid, Waseem El-Huneidi, Amal Hussein

**Affiliations:** 1 College of Medicine, University of Sharjah, Sharjah, ARE; 2 Pharmacology, University of Sharjah, Sharjah, ARE; 3 Family and Community Medicine, University of Sharjah, Sharjah, ARE

**Keywords:** adults, attitudes, dehydration, health public, knowledge, practices, prevention, united arab emirates, water

## Abstract

Introduction

Dehydration may result in many neurological, dermatological, and cardiovascular detrimental effects. The hot humid climate of the United Arab Emirates (UAE) is thought to heavily contribute to daily water loss. No article to this date assesses the public's knowledge about dehydration in the UAE. The aim of this study is to estimate dehydration knowledge level and its determinants among adults in the UAE.

Methods

This cross-sectional study used an online trilingual self-administered questionnaire shared via social media during the first quarter of 2022. Using a non-probability volunteer sampling method, Arabic-, English-, or Indian language-speaking adults aged 18-60 living in the UAE were included. Demographics, associated factors, knowledge level, attitudes, and practices-related data were collected, and a knowledge score was calculated.

Results

Four hundred and eighty-five participants were included, of which 197 (59.4%) were non-locals. The mean knowledge score of all participants on a scale of 0-20 was 11.4 (SD=2.7). Local participants had a lower mean score than non-locals (11.1 and 11.6, respectively; p=0.02). Two hundred and eighty-eight (60%) of the participants don't know the daily minimum recommended water intake. Seizures, coma, and pimples were least to be correctly identified as complications of water deprivation. In contrast to healthy participants, diseased participants seemed to have lower knowledge about dehydration (11.3 and 11.5, respectively; p=0.03); however, unexpectedly, they also reported lower reported incidence of both dehydration (OR=1.94; CI (1.16-3.23); p=0.01) and hospitalization due to dehydration (OR=8.93; CI (1.17-66.7); p=0.01).

Conclusion

Knowledge about dehydration of the majority of UAE's adult population was above average. Awareness campaigns should target the whole community specifically at-risk populations.

## Introduction

Dehydration, characterized by an imbalance between fluid intake and loss, is a significant concern among adults. Despite its importance, no universally accepted definition exists, leading to inconsistencies in reported incidence [1]. Studies indicate that dehydration rates vary by gender, with women being more susceptible than men [2]. While daily water intake recommendations depend on factors such as physical activity, temperature, age, and diet, general guidelines suggest 3.7 liters for men and 2.7 liters for women [3].

Several risk factors for dehydration have been identified, including advanced age, dementia, infections, comorbidities, certain medications, immobility, and intense physical activity [4]. Severe dehydration can result in adverse effects such as tachycardia, hypotension, and, in rare cases, syncope [5]. Inadequate water intake is also associated with complications like constipation, reduced skin turgor, dry skin, and eczema [6,7]. Research shows that even a 2% loss of body weight due to dehydration can impair performance, while greater losses may lead to headaches, irritability, and life-threatening consequences [8].

In the United Arab Emirates (UAE) and the broader Middle East and North Africa (MENA) region, the hot and humid climate exacerbates water loss [9]. Research among UAE workers indicates significant variability in dehydration levels, with construction workers being particularly at risk [10]. Among university students in the UAE, up to 41.3% were found to be hypohydrated with medical students having higher knowledge than non-medical students [9]. 

Public knowledge plays a crucial role in shaping behaviors that prevent and manage dehydration. While studies have examined awareness in high-risk groups such as athletes, students, and caregivers, limited research exists on the general adult population [11-17]. For example, studies in China and Saudi Arabia revealed inadequate knowledge of daily water intake and dehydration symptoms, though these studies often failed to explore associations with physical activity and faced limitations such as selection bias [18,19]. An old report from Kuwait reports that while the majority has good knowledge about the importance of water consumption, the majority drink less than the recommended amount [20]. To date, no prior studies locally exist that investigated this aspect. The findings of this study will empower local decision-making, fuel the national statistical reserve of data and knowledge, and provide answers to relevant questions.

Currently, no validated tool exists to assess dehydration knowledge comprehensively, and prior studies have relied on self-developed questionnaires [18,19]. Similarly, using a self-developed questionnaire, this study seeks to address these gaps by estimating the level of dehydration knowledge among adults in the UAE and identifying factors influencing their awareness and practices.

## Materials and methods

Study design and data collection

A cross-sectional study was conducted to estimate the prevalence of dehydration knowledge, attitudes, and practices among adults in the UAE. A trilingual, self-administered online questionnaire (see Appendices) using Google Forms was distributed via various social media platforms, including WhatsApp, Instagram, Twitter, email, and SMS. Data collection took place from February 24, 2022, to March 23, 2022.

The study included any adult (aged 18 years or more) residing in the UAE who speaks Arabic, English, or Hindi. Exclusion criteria included age younger than 18, residing outside UAE, and not being a speaker of Arabic, English, or Hindi. 

The questionnaire was developed by adopting relevant concepts from a similar study by Shaheen et al. [19], as no standardized tool exists to assess dehydration knowledge.

Questionnaire development and validation

The questionnaire consisted of 25 questions, divided into four sections. Section A focused on demographics, including variables such as gender, age, nationality, education level, occupation, and income. Section B covered associations related to daily consumption of water, tea, and juice, as well as the presence of comorbidities, outdoor exposure, and accessibility to water. Section C assessed knowledge through five key questions on daily water requirements, factors associated with dehydration (eight statements), symptoms (13 symptoms), complications (seven complications), and causes of water loss (eight causes). These questions were measured using Likert scales and multiple-choice formats. Finally, Section D explored participants' attitudes and practices regarding dehydration prevention and management strategies.

The questionnaire, originally developed in English, was translated into Arabic and Hindi with the assistance of native speakers. Pilot testing on 20 participants (not included in the final population) was conducted on a small sample from the target population to ensure clarity, comprehensibility, and content validity across all three language versions. Feedback from pilot testing resulted in minor adjustments to improve clarity.

Sampling method and sample size calculation

A non-probability volunteer sampling method was used to recruit participants. The sample size was calculated using the formula $n=\frac{Z^2 P(1-P)}{ME^2}$ where Z is 1.960 (for a 95% confidence level), P is 0.5 (assumed prevalence due to lack of available data), and ME is 0.05 (margin of error). The estimated sample size was calculated as follows: $n=\frac{(1.960)^2 \times 0.5(1-0.5)}{(0.05)^2}=384.16$. Allowing for a 20% non-response rate, the final sample size was increased to 480 participants.

Statistical analysis

Data from the three language versions were compiled and coded in Excel before analysis. Each participant was assigned a unique ID, generated from the last three letters of their first name and mobile number, to detect and remove duplicate entries. Descriptive statistics, including frequencies and percentages, were used to summarize demographic and knowledge-related data. A dehydration knowledge score (KS) was calculated based on responses to five key questions, with correct answers assigned 1 point and incorrect answers assigned 0 points. The KS was adjusted to a weighted total of 20 points to ensure the most relevant knowledge factors were not underrepresented. Specifically, 1 point was allocated to daily water intake knowledge, 4 points to factors associated with dehydration, 5 points to knowledge of dehydration symptoms, 5 points to knowledge of dehydration complications, and 5 points to knowledge of causes of water loss.

Bivariate analysis was performed to assess associations between the KS and demographic characteristics using appropriate parametric tests, including the independent t-test, one-way ANOVA, and Pearson correlation. Non-parametric tests, such as the chi-squared test, Mann-Whitney U test, and Kruskal-Wallis test, were also applied where appropriate. A significance level of p<0.05 was considered statistically significant. All statistical analyses were conducted using IBM SPSS Statistics for Windows, Version 28.0 (Released 2021; IBM Corp., Armonk, New York, United States).

Ethical considerations

Ethical approval for the study was obtained from the Research Ethics Committee of the University of Sharjah (approval number: REC-22-02-16-04-S). Informed consent was obtained from all participants before they completed the questionnaire, ensuring voluntary participation and confidentiality of responses.

## Results

Demographics

A total of 523 responses were collected. After data cleaning to remove duplicates using a unique ID, 485 responses were included in the final analysis. The demographic characteristics of the participants are summarized in Table 1. The majority of participants were female (332, 68.5%) and young adults (378, 77.9%). Approximately half of the participants had a healthy weight, while 179 (40.9%) were classified as overweight or obese. Additionally, 84 (17.3%) reported having underlying comorbidities, with heart disease (35, 37.6%) and gastrointestinal disease (32, 34.4%) being the most common. Table 1 provides a detailed breakdown of demographic characteristics.

**Table 1:** Demographic characteristics of participants (n=485) ^a^39 participants had missing values (n=446) ^b^3 participants had missing values (n=482) ^*^: independent t-test; ^**^: Mann-Whitney U-test; ^***^: Kruskal-Wallis test; ^****^: one-way ANOVA P-values in bold are considered significant

Factors	Frequency	Percentage (%)	P-value
Gender			0.85^*^
Male	153	31.5	
Female	332	68.5	
Age group			0.85^*^
18-30 (young adult)	378	77.9	
31-60 (middle and older adult)	107	22.1	
Nationality			0.02^*^
Local	197	40.6	
Non-local	288	59.4	
Level of education			0.32^**^
Diploma or lower	207	42.7	
Undergraduate or higher	278	57.3	
Occupation^a^			0.08*
Student	253	56.7	
Non-student	193	43.3	
Income group			0.92^***^
Less than 5000 DHS/month	87	17.9	
Between 5000 and 10000 AED/month	93	19.2	
Between 10000 and 20000 AED/month	118	24.3	
More than 20000 AED/month	187	38.6	
BMI^b^			0.30^****^
Underweight	43	8.9	
Healthy weight	242	50.2	
Overweight	126	26.1	
Class 1 obesity	48	10	
Class 2 obesity	17	3.5	
Class 3 obesity	6	1.2	
Presence of comorbidities			<0.001^**^
Present	84	17.3	
Absent	401	82.7	
Comorbidities by type, if present			
Heart disease	35	37.6	0.03^*^
Gastrointestinal disease	32	34.4	
Diabetes	15	16.1	0.02^*^
Renal disease	11	11.8	

Knowledge

Table 2 presents a summary of responses to the 36 knowledge-related variables. The mean KS was 11.4 (SD=2.7), ranging from 1.4 to 19.0. More than half of the participants (291, 60%) were unable to correctly identify the minimum daily recommended water intake. Factors such as sleep, humidity, inconsistent food intake, and exercise were among the least frequently recognized contributors to dehydration.

**Table 2:** Knowledge towards dehydration (n=485)

Knowledge	Frequency	Percentage (%)
Minimum recommended daily water intake		
Correct	194	40
Wrong	291	60
Correctly identified factors related to dehydration		
Drinking fluids reduces dehydration risk	390	80.4
Exposure to hot climates increases dehydration risk	378	77.9
Diarrhea increases dehydration risk	366	75.5
Eating high-water-containing foods reduces dehydration risk	348	71.8
Exercise increases dehydration risk	258	53.2
Inconsistent food intake may cause dehydration	248	51.1
Humidity increases dehydration risk	243	50.1
Lack of sleep may cause dehydration	192	39.6
Correctly identified causes of water loss		
Sweating	408	84.1
Diarrhea	389	80.2
Increased urination	361	74.4
Vomiting	341	70.3
Fever	250	51.5
Stress	237	48.9
Flight travel	75	15.5
Correctly identified dehydration symptoms		
Dry lips	438	90.3
Thirst	414	85.4
Dry tongue	404	83.3
Dizziness	342	70.5
Dark-colored urine	324	66.8
Loss or difficulty in concentration	301	62.1
Light-headedness	294	60.6
Decreased urination	286	59
Fatigue	270	55.7
Rapid breathing	174	35.9
Rapid pulse	166	34.2
Muscle weakness	131	27
Muscle cramps	121	24.9
Dehydration complications		
Headache	400	82.5
Renal failure	289	59.6
Mood changes	267	55.1
Heat stroke	266	54.8
Pimples	193	39.8
Coma	170	35.1
Seizures	158	32.6

Regarding hydration substitutes, 180 (37.1%) of participants agreed or strongly agreed that consuming high-water-content foods could replace drinking water, while 193 (39.8%) disagreed or strongly disagreed, and 112 (23.1%) were neutral.

Flight travel, stress, and fever were among the least identified causes of water loss. Additionally, dehydration symptoms such as rapid breathing (35.9%; n=174), rapid pulse (34.2%; n=166), muscle weakness (27%; n=131), and muscle cramps (24.9%; n=121) were the least recognized. Complications like seizures, coma, and pimples were identified correctly by fewer than half of the participants.

Statistical analysis revealed that KSs were significantly lower among local participants compared to non-locals (mean KS: 11.1 vs. 11.6; p=0.02; independent t-test). Participants with underlying diseases had significantly lower mean KS ranks than healthy individuals (212.2 vs. 249.5; p<0.001; Mann-Whitney U-test). Notably, participants with heart disease or diabetes scored lower (p=0.03 and p=0.02, respectively; independent t-test). Those who reported having been hospitalized for dehydration had lower KS ranks than those who had not (88.4 vs. 108.3; p=0.04; Mann-Whitney U-test).

Associations

Table 3 presents the associations between daily hydration-related behaviors and KSs. The majority of participants (227, 47.1%) reported drinking 3-6 cups of water daily, while only 69 (14.3%) reported consuming more than 10 cups. A significant association was found between the number of daily cups of water consumed and KSs (p<0.001; Kruskal-Wallis test), with participants drinking 7-10 cups having the highest mean KS of 12.3 (SD=3.2).

**Table 3:** Associations related to dehydration (n=485) *: independent t-test; ***: Kruskal-Wallis test; ****: one-way ANOVA P-values in bold are considered significant

Associations	Frequency	Percentage (%)	P-value
Cups of water intake			<0.001^***^
Less than 3 cups	84	17.4	
3-6 cups	227	47.1	
7-10 cups	102	21.2	
More than 10 cups	69	14.3	
Cups of juice/soft drink intake			0.72^***^
None	72	14.9	
1 cup	325	67.4	
2 or more cups	85	17.6	
Cups of coffee/tea intake			0.89^***^
None	27	5.6	
1 cup	252	52.3	
2 or more cups	203	42.1	
Hours of weekly workout (activity)			0.373^****^
None	167	34.6	
Less than 3 hours	195	40.5	
More than 3 hours	120	24.9	
Hours of daily sun exposure			0.01^***^
None	90	18.7	
Less than 1 hour	236	49	
More than 1 hour	156	32.4	
Hours of daily outdoor work if applicable			0.21^*^
Less than 5 hours	298	79	
More than 5 hours	79	21	
Ability to access water in the workplace if applicable (if work involves outdoors work for more than 5 hours)			0.81^*^
Yes	64	79	
No	17	21	

Most participants reported consuming at least one cup of coffee or tea (445, 94.4%) compared to juice or soft drinks (410, 85%). However, the frequency of coffee/tea and juice/soft drink intake showed no significant association with KS (p=0.89 and p=0.72, respectively).

Regarding physical activity, 167 (34.6%) of participants reported no regular exercise, and 90 (18.7%) reported no daily sun exposure. Participants with less than one hour of sun exposure had a significantly higher mean KS (11.7; SD=2.7; p=0.01). Among those who worked outdoors for more than five hours daily, 64 (79%) reported having easy access to water at their workplace.

Attitudes and practices

Table 4 presents the attitudes and practices adopted by participants to prevent and manage dehydration. The majority of participants (369, 76.1%) reported drinking plenty of fluids as a preventive measure, while 367 (75.7%) packed enough water, and 249 (51.3%) set reminders to drink water. Less commonly adopted practices included avoiding sun exposure (114, 23.5%), avoiding salty foods (123, 25.4%), and wearing light clothes (104, 21.4%). All practices, except drinking plenty of fluids, were significantly associated with higher KSs.

**Table 4:** Attitudes and practices towards dehydration (n=485) *: independent t-test; **: Mann-Whitney U-test P-values in bold are considered significant

Attitudes and practices	Frequency	Percentage (%)	P-value
To prevent dehydration, I…			
Drink plenty of fluids	369	76.1	0.14^*^
Pack enough water	367	75.7	<0.001^*^
Set a reminder to drink water	249	51.3	0.002^*^
Avoid exercise in hot climates	189	39	0.002^*^
Eat foods with high water content	138	28.5	<0.001^*^
Avoid salty food	123	25.4	<0.001^*^
Avoid sun exposure	114	23.5	<0.001^*^
Use skin moisturizers	111	22.9	<0.001^*^
Wear light clothes	104	21.4	<0.001^*^
To manage dehydration, I…			
Drink a lot of bottled water	410	84.5	0.15^*^
Go to the nearest emergency department	148	30.5	0.90^*^
Drink tonic water (i.e., water with salt)	110	22.7	0.62^*^
Have you ever experienced dehydration?			0.92^**^
Yes	206	42.5	
No	279	57.5	
Symptoms experienced when dehydrated, if applicable			
Thirst	150	72.8	0.69^*^
Dry lips	160	77.7	0.16^*^
Dry tongue	129	62.6	0.65^*^
Headache	145	70.4	0.29^*^
Dizziness	122	59.2	0.96^*^
Light-headedness	109	52.9	0.76^*^
Rapid breathing	49	23.8	0.90^*^
Rapid pulse	64	31.1	0.43^*^
Decreased urination	98	47.6	0.71^*^
Dark-colored urine	123	59.7	0.95^*^
Fatigue	96	46.6	0.87^*^
Muscle cramps	42	20.4	0.60^*^
Muscle weakness	60	29.1	0.71^*^
Pimples	95	46.1	0.36^*^
Lack of focus	128	61.8	0.65^*^
Loss or difficulty in concentration	111	53.9	0.98^*^
Mood changes	85	41.3	0.32^*^
When you experienced dehydration, did you need hospitalization?			0.04^**^
Yes	50	24.3	
No	156	75.7	

Regarding dehydration management, 410 (84.5%) reported drinking bottled water, 148 (30.5%) sought emergency care, and 110 (22.7%) consumed tonic water (salted water).

Self-reported dehydration incidence (SRDI) was observed in 206 (42.5%) of participants, with 50 (24.3%) requiring hospitalization. Common dehydration symptoms included dry lips (160, 77.7%), thirst (150, 72.8%), and headache (145, 70.4%). Less frequently reported symptoms included muscle cramps (42, 20.4%) and rapid breathing (49, 23.8%).

Bivariate analysis showed significant associations between SRDI and factors such as being a young adult (OR=2.11; 95% CI: 1.32-3.37; p=0.001; chi-squared test), being local (OR=1.54; 95% CI: 1.07-2.22; p=0.02; chi-squared test), and being a student (OR=1.74; 95% CI: 1.18-2.56; p=0.005; chi-squared test). Interestingly, participants drinking fewer cups of water daily had lower SRDI compared to those drinking more than 10 cups, and participants with comorbidities had significantly lower SRDI (OR=1.94; 95% CI: 1.16-3.23; p=0.01; chi-squared test).

Self-Reported Dehydration Hospitalization Incidence (SRDHI)

Factors associated with higher SRDHI included being female (OR=3.77; 95% CI: 1.51-9.42; p=0.003), experiencing rapid pulse (OR=2.39; 95% CI: 1.23-4.63; p=0.009; chi-squared test), and dizziness (OR=2.38; 95% CI: 1.17-4.82; p=0.02; chi-squared test). Lower SRDHI was observed among participants with comorbidities (OR=8.93; 95% CI: 1.17-66.7; p=0.01; chi-squared test) and those who wore light clothing to prevent dehydration (OR=2.90; 95% CI: 1.07-7.81; p=0.03; chi-squared test).

## Discussion

This study analyzes three main outcomes: KS, SRDI, and SRDHI. Knowledge was assessed using the KS, which provided an overall computable statistic. The average KS among adults in the UAE was 11.4 (SD=2.7) out of 20.0, indicating an above-average knowledge level. This is comparable to findings from Saudi Arabia, where participants demonstrated good knowledge regarding dehydration symptoms and water intake recommendations [19].

However, despite the above-average KS, only 69 (14.3%) of participants reported drinking more than the recommended daily water intake, and 291 (60%) were unaware of the minimum recommended intake. This finding contrasts with data from Saudi Arabia, where over two-thirds of the population reported meeting or exceeding the daily intake recommendations [19]. Similar to previous studies, participants demonstrated limited awareness of less common dehydration complications, such as seizures, coma, and pimples [19,21-22].

KSs varied significantly based on nationality, presence of comorbidities, daily water intake, daily sun exposure, SRDHI, and prevention practices. Unlike findings by Shaheen et al. [19], our study did not identify significant differences in KSs across genders. Consistent with prior research [21], no significant variation in KSs was found across different educational levels. Participants who had been hospitalized for dehydration had lower KS ranks, whereas prior studies have shown that hospitalized patients tend to drink more water [21].

Dehydration-related hospitalizations present a substantial economic burden, with an estimated cost of $5.5 billion in the United States in 2004 [23]. The SRDI significantly varied with age, occupation, nationality, comorbidities, daily water intake, and the practice of setting reminders to drink water. Unlike findings from the United States, where income and BMI were associated with SRDI, our study did not identify a significant correlation [24,25].

Unexpectedly, participants who consumed fewer cups of water daily and those who did not set reminders reported lower SRDI. One potential solution to address this gap is hydration game reminders, which have been shown to improve adherence to regular water intake [26,27].

The SRDHI varied significantly by gender, presence of comorbidities, preventive practices (light clothing), and dehydration symptoms (dizziness and rapid pulse). Female participants are generally more vulnerable to dehydration, which may explain their higher SRDHI in this study [2,4]. Clothing plays an essential role in heat regulation and water balance, with participants who wore light clothing experiencing significantly lower SRDHI rates [28]. Moreover, the significant association between SRDHI and symptoms such as rapid pulse and dizziness aligns with previous studies, which reported similar post-admission findings in dehydrated patients [29,30].

The accuracy of our findings may have been affected by self-selection bias, as the study relied entirely on self-reported data. Additionally, the precise determination of SRDI and SRDHI was limited by the nature of the data collection instrument being reliant on patient-reported data rather than medical records. Due to the fact that data collection was concurrent with the restrictions of the COVID-19 pandemic, the methodology of data collection was limited to the online questionnaire. However, this is a popular method both prior to and post the pandemic, and we tried to reduce the risk of duplicate or false entries by assigning each participant a unique ID and then filtering out any duplicates. Nevertheless, this study is the first national investigation addressing dehydration knowledge and behaviors among the adult population in the UAE. Future research should consider longitudinal studies to assess knowledge and behavior changes over time. Additionally, future research should utilize a more robust probability sampling and determine clinically if possible the level and presence of dehydration in the public. 

## Conclusions

Overall, the majority of adults in the UAE demonstrated above-average knowledge about dehydration. However, individuals with comorbidities exhibited lower knowledge levels compared to healthy individuals, yet they also reported lower incidence rates of both dehydration and hospitalization. Targeted awareness campaigns should be designed to educate the entire community, with a particular focus on at-risk populations.

## Appendices

Below is the original English version of the questionnaire used in the study in table format. Kindly contact us for Arabic or Hindi versions if needed. 

**Table 5:** English version of the questionnaire

Section	Question/instruction	Options/format
Initial inclusion and exclusion questions	If the response to any is "No", then participants are directed to the "Thank You" page	
	Please choose a language to fill the survey with:	English
		Arabic
		Hindi
	Are you currently living in the UAE? (Note: living in the UAE is residing in any emirate for purposes other than tourism)	Yes
		No
	Are you between the ages 18 and 60?	Yes
		No
	Please write the last three digits of your phone number:	Open-ended
	Please write the first three letters of your first name:	Open-ended
Section A: demographics	Please specify your gender:	Male
		Female
	Please specify the age group you belong to:	18-30 (young adult)
		31-50 (middle-aged)
		>50 (older adults)
	Please specify where you originally came from:	Drop-down list of all countries listed in the UN
	Please specify what is your highest level of education:	No education
		Elementary school level
		Middle school level
		Secondary/high school level
		Diploma level
		Undergraduate level (bachelor's)
		Post-graduate (master's or higher)
	Please specify what your occupation is:	Open-ended
	Please specify your family's monthly income group:	No income
		<2,500 DHS/month
		2,500-5,000 DHS/month
		5,000-10,000 DHS/month
		10,000-20,000 DHS/month
		>20,000 DHS/month
Section B: associations	Please write your height in cm:	Open-ended
	Please write your weight in kg:	Open-ended
	How many cups of water do you drink per day? This doesn't include other sources of water (e.g., juice, coffee, soft drinks, etc.) or water-containing foods. (Refer to the diagram for cup amount in mL)	<3
		3-6
		7-10
		11-14
		15-18
		>18 (specify number)
	How many cups of coffee or tea do you drink per day? (Refer to the diagram for cup amount in mL)	<1
		2-3
		>3 (specify number)
	How many cups of juice or soft drinks do you drink per day? (Refer to the diagram for cup amount in mL)	<1
		2-3
		>3 (specify number)
	Do you have any of the following diseases? (Choose all that apply)	Heart disease (including hypertension, heart failure, arrhythmia, etc.)
		Diabetes
		Renal diseases (e.g., kidney stones, chronic renal failure)
		Gastrointestinal diseases
	Please specify how many hours of workout/activity (e.g., walking, running, jogging, swimming) you do weekly:	None
		<3 hours
		3-5 hours
		6-8 hours
		>8 hours
	If applicable, does your job include mostly outdoor work (more than 5 hours daily)?	Yes
		No
	If the answer to the above is "Yes", does your employer provide easily accessible water (adequate cost and distance)?	Yes
		No
	On average, how much time do you spend in the sun per day regardless of the reason?	None
		<1 hour
		1-2 hours
		2-3 hours
		3-4 hours
		>4 hours
Section C: knowledge	How much do you think is the minimum amount of recommended water intake per day for an average 70 kg adult?	1 liter
		2 liters
		3 liters
		4 liters
	Please select the degree to which you agree with the following statements:	Likert scale (Strongly Disagree to Strongly Agree)
	Drinking fluids (e.g., water, juices) reduces the risk of dehydration	
	I can become dehydrated if I don't get enough sleep	
	I can become dehydrated if my food intake is inconsistent	
	Hot climate exposure increases the risk of dehydration	
	Humidity increases the risk of dehydration	
	Exercise increases the risk of dehydration	
	Diarrhea increases the risk of dehydration	
	Eating high-water-content foods (e.g., watermelons, oranges, cucumber, etc.) reduces the risk of dehydration	
	Eating high-water-content foods (e.g., watermelons, oranges, apples, etc.) can be an adequate alternative to water	
	Please choose all that you think could cause water loss:	Sweating
		Fever
		Vomiting
		Diarrhea
		Increased urination
		Flight travel
		Stress
		Coffee
	In your opinion, choose all that can be a symptom of dehydration:	Thirst
		Dry lips
		Dry tongue
		Dizziness
		Light-headedness
		Rapid breathing
		Rapid pulse
		Decreased urination
		Dark-colored urine
		Fatigue
		Muscle cramps
		Muscle weakness
		Loss or difficulty in concentration
	In your opinion, choose all that can be a complication of dehydration:	Headache
		Pimples
		Mood changes
		Coma
		Seizures
		Renal failure
		Heat stroke
Section D: attitudes and practices	Please select all that you do to prevent dehydration:	Drink plenty of fluids
		Avoid exercise in hot climates
		Avoid sun exposure
		Pack enough water
		Set reminders to drink water
		Avoid salty food
		Wear light clothes
		Use skin moisturizers
		Eat foods with high water content (e.g., watermelons, cucumbers)
		Others (specify):
	Please select all that you do to manage dehydration:	Drink a lot of bottled water
		Drink water with salt (tonic water)
		Go to the nearest emergency department
		Others (specify):
	Have you ever experienced dehydration?	Yes
		No
	If the answer to the above is "Yes", choose the symptoms you experienced:	Thirst
		Dry lips
		Dry tongue
		Headache
		Dizziness
		Light-headedness
		Rapid breathing
		Rapid pulse
		Decreased urination
		Dark-colored urine
		Fatigue
		Muscle cramps
		Muscle weakness
		Pimples
		Lack of focus
		Loss or difficulty in concentration
		Mood changes
	If the answer to the above is "Yes", did you need hospitalization?	Yes
		No
